# Editorial for the Molecular Genetics and Genomics of Metabolic Disorders in Cardiovascular and Cerebrovascular Diseases Special Issue: June 2023

**DOI:** 10.3390/genes14081568

**Published:** 2023-08-01

**Authors:** Yuanyuan Zhao, Xin Tu

**Affiliations:** 1 Institute of Organ Transplantation, Tongji Hospital, Tongji Medical College, Huazhong University of Science and Technology/Key Laboratory of Organ Transplantation, Ministry of Education/NHC Key Laboratory of Organ Transplantation, Key Laboratory of Organ Transplantation, Chinese Academy of Medical Sciences, Wuhan 430074, China; 2 Key Laboratory of Molecular Biophysics of the Ministry of Education, Center for Human Genome Research, College of Life Science and Technology, Huazhong University of Science and Technology, Wuhan 430074, China

Cardiovascular and cerebrovascular diseases are the leading causes of the mortality of humans in the 21st century. Furthermore, the prevalence and absolute number of cardiovascular and cerebrovascular diseases will continue to increase because of the prolonged survival of patients and the aging population. The underlying pathology is atheromatous vascular disease, resulting in coronary artery disease (CAD), cerebrovascular disease, and peripheral vascular disease, in addition to the subsequent development of heart failure, cardiac arrhythmias, and other related disorders [1]. Cerebrovascular disease also includes hemorrhagic and ischemic stroke. The risk factors for cardiovascular and cerebrovascular disorders were discovered several decades ago and recognized ever since. The main risk factors usually include heredity (gene mutations and polymorphisms, epigenetic regulation, and cellular regulation, such as autophagy), and modifiable as well as environmental risk factors (hypertension, high levels of low-density lipoprotein (LDL) cholesterol, smoking, alcohol, diabetes, and abdominal obesity). Furthermore, lack of exercise and psychosocial disorders are also associated with cardiovascular and cerebrovascular diseases; however, the molecular genetics and causality of modifiable disorders in cardiovascular and cerebrovascular diseases remain unclear, and the understanding of the molecular genetics and genomics of this area is still a major challenge at present. This Special Issue contains seven original research articles and two review papers that further our collective knowledge of the etiology of cardiovascular and cerebrovascular diseases, as well as the genetic and modifiable risk factors underlying the diseases.

Heredity is one of the most important risk factors for cardiovascular and cerebrovascular diseases, and a large number of studies have demonstrated that gene mutations and polymorphisms are associated with cardiovascular and cerebrovascular disorders. In this Special Issue, Zhou et al. [2] investigate the possible mechanisms of mediator complex subunit 12 (human: *MED12*; mouse: *Med12*) involvement in aortic dissection (AD). In this study, the authors examine the expression of the MED12 protein in the aortic tissues of AD patients as well as AD mice, and verify that MED12 is deficient in AD patients/mice. Using mouse aortic smooth muscle cells (MOVASs), they observe that the downregulation of Med12 can inhibit the proliferation of MOVASs and promote senescence. Finally, they demonstrate that Med12 can inhibit the TGFβ1 nonclassical signaling pathway, while TGFβ1 inhibits the phenotype transformation and proliferation of MOVASs by inhibiting Med12 synthesis. AD is a life-threatening aortic disease, and the mortality rate of AD is 35.8%. Previous molecular genetic studies have demonstrated that *MED12* gene mutations can lead to inherited diseases, and this study presents the *MED12* gene as a potential target for the prevention and treatment of AD. In another study in this Special Issue, Wang et al. [3] pay attention to the pathogenic gene *FBN1* in Marfan syndrome (MFS), which is a highly penetrant and lethal autosomal dominant genetic disorder. Gene *FBN1*, which encodes the extracellular matrix glycoprotein fibrillin-1, is the main pathogenic gene of MFS. In this study, they present a novel splice-altering pathogenic mutation (c.8051+1G>C) in the splice site of exon 64 of gene *FBN1* that causes MFS. Subsequent splicing analyses reveal that the mutation can cause both the complete and partial (two splicing products) deletion of exon 64, which lead to pretermination and a shortened fibrillin-1 protein. In addition, they also systematically summarize previous reported transcriptional studies on 258 pathogenic splice-altering mutations in the *FBN1* gene. This study discusses a specific genetic condition where one splice-altering mutation can cause multiple transcripts.

Apart from the main pathogenic genes, a huge number of gene variants and polymorphisms contribute to CVD. In this Special Issue, Ali et al. [4] investigate twenty-two public cDNA Affymetrix datasets to discover genes associated with hypertension (HT). In total, they rank seven HT-related genes, including *ADM*, *ANGPTL4*, *USP8*, *EDN*, *NFIL3*, *MSR1*, and *CEBPD*. In addition, SP1, KLF7, and STAT1 are considered transcriptional factors associated with regulatory mechanisms. In another study, Balcerzyk-Mati´c et al. [5] focus on the genetic factors that influence the survival of patients with CAD. Their study includes 276 patients hospitalized due to CAD, with a medical history and genotypic results of 29 polymorphisms. Finally, they identify that *AGT*, *ABCA1*, and *CYBA* gene polymorphisms can influence the risk of death in patients with CAD. In addition, Susilo et al. [6] pay attention to the effect of angiotensin-converting enzyme (ACE) insertion/deletion (I/D) polymorphisms on atherosclerotic cardiovascular disease and cardiovascular mortality risk in nonhemodialyzed chronic kidney disease (CKD). These studies indicate that CVDs are fairly complicated diseases involving polygenic interaction. Furthermore, Schmidtke [7] presents a snapshot of the past twenty-five years’ work in hereditary hemochromatosis (HH) based on genotyping screening. In fact, HH is an autosomal recessive disorder characterized by increased iron absorption and iron overload, affecting multiple organs. In their review, Schmidtke summarizes the biochemical and clinical penetrance of HH, discusses the societal response to population screening for HH, and suggests a few professional recommendations using selected examples from the literature.

In addition to genetic factors, modifiable and environmental risk factors also play key roles in cardiovascular and cerebrovascular diseases. This Special Issue also collects some research on this aspect. Zhao et al. [8] provide a review paper that summarizes potential microRNAs (miRNAs) and miRNA-related regulatory mechanisms for cardiac fibrosis and discusses the therapeutic strategies of cardiac fibrosis through the modulation of miRNAs. This review helps us to understand that noncoding RNAs have a crucial role in the pathological development of cardiovascular disorders through epigenetic regulation. In addition, cellular regulation can also influence these disorders. Diao et al. [9] focus on the role of B cell lymphoma 2-associated athanogene (BAG3) in atherosclerosis. They use ApoE^−/−^ mice to overexpress BAG3 and finally verify the fact that BAG3 alleviates atherosclerosis by inhibiting the endothelial-to-mesenchymal transition via autophagy activation. These studies provide a new angle that helps us understand multiple regulation mechanisms in CVDs.

Finally, in order to discover novel environmental risk factors associated with CVD, Zhou et al. [10] perform two-sample Mendelian randomization (MR) studies to assess the association between genetic liability for periodontal diseases (dental caries and periodontitis) and major CVDs, including CAD, heart failure (HF), atrial fibrillation (AF), and stroke based on large-scale genome-wide association studies (GWASs). Their study does not provide evidence for dental caries and periodontitis as causes of cardiovascular diseases; however, through it, we can realize that MR is a burgeoning kind of data analysis method that is mainly used in epidemiological etiology inference.

In summary, the research and review papers in this Special Issue cover a range of topics and provide comprehensive insights with which to direct future cardiovascular and cerebrovascular disease research. A growing number of heredity, modifiable, and environmental risk factors have been found to be associated with these disorders. It is important that, although the collected papers in this Special Issue do not discuss the risk factor of gender in CVDs, the impact of gender on CVD risk is well recognized. Inherited variations in sex chromosomes, hormonal differences, and health risk behaviors all may contribute to some extent to this sex difference in CVDs. In conclusion, we hope that this Special Issue will help researchers search for additional genetic and environmental associations that will help refine our understanding of the etiology of complex diseases.

## Data Availability

Not applicable.
